# Supplementary material for: Anthropometric, Physiological, and Psychological Variables That Determine the Elite Pistol Performance of Women
Source: Int J Environ Res Public Health. 2022 Jan 19;19(3):1102. doi: 10.3390/ijerph19031102 (PMC8834022; doi:10.3390/ijerph19031102)
Supplement: Supplementary file 1 [file ijerph-19-01102-s001.zip › ijerph-1510646-supplementaryS1.pdf]

| Q. | **Sport anxiety scale (SAS)** | Not At All | Somewhat | Moderately So | Very Much So |
|---|---|---|---|---|---|
| 1 | I feel nervous | 1 | 2 | 3 | 4 |
| 2 | During competition, I find myself thinking about unrelated things | 1 | 2 | 3 | 4 |
| 3 | I have self-doubts | 1 | 2 | 3 | 4 |
| 4 | My body feels tense | 1 | 2 | 3 | 4 |
| 5 | I am concerned that I may not do as well in competition as I could | 1 | 2 | 3 | 4 |
| 6 | My mind wanders during sport competition | 1 | 2 | 3 | 4 |
| 7 | While performing, I often do not pay attention to what's going on | 1 | 2 | 3 | 4 |
| 8 | I feel tense in my stomach | 1 | 2 | 3 | 4 |
| 9 | Thoughts of doing poorly interfere with my concentration during | 1 | 2 | 3 | 4 |
| 10 | I'm concerned about choking under pressure | 1 | 2 | 3 | 4 |
| 11 | My heart races | 1 | 2 | 3 | 4 |

| | | | | | |
|---|---|---|---|---|---|
| 12 | I feel my stomach sinking | 1 | 2 | 3 | 4 |
| 13 | I'm concerned about performing poorly | 1 | 2 | 3 | 4 |
| 14 | I have lapses of concentration during competition because of nervousness | 1 | 2 | 3 | 4 |
| 15 | I sometimes find myself trembling before or during a competitive event | 1 | 2 | 3 | 4 |
| 16 | I'm worried about reaching my goal | 1 | 2 | 3 | 4 |
| 17 | My body feels tight | 1 | 2 | 3 | 4 |
| 18 | I'm concerned that others will be disappointed in my performance | 1 | 2 | 3 | 4 |
| 19 | My stomach gets upset before or during a competitive event | 1 | 2 | 3 | 4 |
| 20 | I'm concerned I won't be able to concentrate | 1 | 2 | 3 | 4 |
| 21 | My heart pounds before competition | 1 | 2 | 3 | 4 |

## Sport Motivation Scale-6 (Sms-6) questionnaire

Using the scale below, please indicate to what extent each of the following items corresponds to one of the reasons for which you are presently practicing your sport.

Does not correspond at al = 1

Corresponds a little = 2-3

Corresponds moderately= 4

Corresponds a lot= 5

Corresponds exactly= 6-7

1. For the excitement I feel when I am really involved in the activity 1 2 34567
2. Because it's part of the way in which I've chosen to live my life 1 2 34567
3. Because it is a good way to learn lots of things which could be useful to me in other areas of my life 1234567
4. Because it allows me to be well regarded by people that I know 1 2 34567
5. I don't know anymore; I have the impression of being incapable of succeeding in this sport 1234567
6. Because I feel a lot of personal satisfaction while mastering certain difficult training techniques 1234567
7. Because it is absolutely necessary to do sports if one wants to be in shape 1234567
8. Because it is one of the best ways I have chosen to develop other aspects of my life 1234567
9. Because it is an extension of me 1 2 34567
10. Because I must do sports to feel good about myself 1 2 34567
11. For the prestige of being an athlete 1 2 34567
12. I don't know if I want to continue to invest my time and effort as much in my sport anymore 1234567
13. Because participation in my sport is consistent with my deepest principles 1234567
14. For the satisfaction I experience while I am perfecting my abilities 1 2 34567
15. Because it is one of the best ways to maintain good relationships with my friends 1234567
16. Because I would feel bad if I was not taking time to do it 1 2 34567
17. It is not clear to me anymore; I don't really think my place is in sport 1 2 34567
18. For the pleasure of discovering new performance strategies 1 2 34567
19. For the material and/or social benefits of being an athlete 1 2 34567
20. Because training hard will improve my performance 1 2 34567
21. Because participation in my sport is an integral part of my life 1 2 34567

22. I don’t seem to be enjoying my sport as much as I previously did 1 2 34567

23. Because I must do sports regularly 1 2 3 4 5 6 7

24. To show others how good I am at my sport 1 2 3 4 5 6 7

# Athletic Coping Skills Inventory-28 (ACSI-28) questionnaire

| | 0 | 1 | 2 | 3 |
|---|---|---|---|---|
| | Almost never | sometimes | often | almost always |

1. On a daily or weekly basis, I set very specific goals for myself that guide what I do.

2. I get the most out of my talent and skill.
3. When a coach or manager tells me how to correct a mistake I've made, I tend to take it personally and feel upset.

4. When I'm playing sports, I can focus my attention and block out distractions.

5. I remain positive and enthusiastic during competition, no matter how badly things are going.

6. I tend to play better under pressure because I think more clearly.

7. I worry quite a bit about what others think of my performance.

8. I tend to do lots of planning about how to reach my goals.

9. I feel confident that I will play well.
10. When a coach or manager criticizes me, I become upset rather than feel helped.

11. It is easy for me to keep distracting thoughts from interfering with something I am watching or listening to.

12. I put a lot of pressure on myself by worrying about how I will perform.

13. I set my own performance goals for each practice.
14. I don't have to be pushed to practice or play hard; I give 100%.

15. If a coach criticizes or yells at me, I correct the mistake without getting upset about it.

16. I handle unexpected situations in my sport very well.

17. When things are going badly, I tell myself to keep calm, and this works for me.

18. The more pressure there is during a game, the more I enjoy it.

19. While competing, I worry about making mistakes or failing to come through.

20. I have my own game plan worked out in my head long before the game begins.

21. When I feel myself getting too tense, I can quickly relax my body and calm myself.

22. To me, pressure situations are challenges that I welcome.

23. I think about and imagine what will happen if I fail or screw up.

24. I maintain emotional control regardless of how things are going for me.

25. It is easy for me to direct my attention and focus on a single object or person.

26. When I fail to reach my goals, it makes me try even harder.

27. I improve my skills by listening carefully to advice and instruction from coaches and managers.

28. I make fewer mistakes when the pressure is on because I concentrate better.

# (TSCI questionnaire) Trait Sport Confidence Inventory

**Activity 2.3: Assessing Sport Confidence: How Confident Are You?**

Instructions: Think about how self-confident you are when you compete in sport.
Answer the following questions based on how confident you generally feel when you compete in your sport. Please try to determine how you really feel, not how you would like to feel. Compare your self-confidence level to the self-confidence level of the most self-confident athlete you know. Your answers will be kept confidential.
Scale: 1 = lowest; 5 = medium; 9 = highest (circle number from 1 to 9)

1. Compare your confidence in your ability to execute the skills necessary to be successful to the most confident athlete you know.

| Low | | | | Medium | | | High | |
|---|---|---|---|---|---|---|---|---|
| 1 | 2 | 3 | 4 | 5 | 6 | 7 | 8 | 9 |

2. Compare your confidence in your ability to make critical decisions during competition to the most confident athlete you know.

| Low | | | | Medium | | | High | |
|---|---|---|---|---|---|---|---|---|
| 1 | 2 | 3 | 4 | 5 | 6 | 7 | 8 | 9 |

3. Compare your confidence in your ability to perform under pressure to the most confident athlete you know.

| Low | | | | Medium | | | High | |
|---|---|---|---|---|---|---|---|---|
| 1 | 2 | 3 | 4 | 5 | 6 | 7 | 8 | 9 |

4. Compare your confidence in your ability to execute successful strategy to the most confident athlete you know.

| Low | | | | Medium | | | High | |
|---|---|---|---|---|---|---|---|---|
| 1 | 2 | 3 | 4 | 5 | 6 | 7 | 8 | 9 |

5. Compare your confidence in your ability to concentrate well enough to be successful to the most confident athlete you know.

| Low | | | | Medium | | | High | |
|---|---|---|---|---|---|---|---|---|
| 1 | 2 | 3 | 4 | 5 | 6 | 7 | 8 | 9 |

6. Compare your confidence in your ability to adapt to different game situations and still be successful to the most confident athlete you know.

| Low | | | | Medium | | | High | |
|---|---|---|---|---|---|---|---|---|
| 1 | 2 | 3 | 4 | 5 | 6 | 7 | 8 | 9 |

7. Compare your confidence in your ability to achieve your competitive goals to the most confident athlete you know.

| Low | | | | Medium | | | High | |
|---|---|---|---|---|---|---|---|---|
| 1 | 2 | 3 | 4 | 5 | 6 | 7 | 8 | 9 |

8. Compare your confidence in your ability to be successful to the most confident athlete you know.

| Low | | | | Medium | | | High | |
|---|---|---|---|---|---|---|---|---|
| 1 | 2 | 3 | 4 | 5 | 6 | 7 | 8 | 9 |

9. Compare your confidence in your ability to be consistently successful to the most confident athlete you know.

| Low | | | | Medium | | | High | |
|---|---|---|---|---|---|---|---|---|
| 1 | 2 | 3 | 4 | 5 | 6 | 7 | 8 | 9 |

10. Compare your confidence in your ability to think and respond successfully during competition to the most confident athlete you know.

| Low | | | | Medium | | | High | |
|---|---|---|---|---|---|---|---|---|
| 1 | 2 | 3 | 4 | 5 | 6 | 7 | 8 | 9 |

11. Compare your confidence in your ability to meet the challenge of competition to the most confident athlete you know.

| Low | | | | Medium | | | High | |
|---|---|---|---|---|---|---|---|---|
| 1 | 2 | 3 | 4 | 5 | 6 | 7 | 8 | 9 |

12. Compare your confidence in your ability to be successful even when the odds are against you to the most confident athlete you know.

| Low | | | | Medium | | | High | |
|---|---|---|---|---|---|---|---|---|
| 1 | 2 | 3 | 4 | 5 | 6 | 7 | 8 | 9 |

13. Compare your confidence in your ability to bounce back from performing poorly and be successful to the most confident athlete you know.

| Low | | | | Medium | | | High | |
|---|---|---|---|---|---|---|---|---|
| 1 | 2 | 3 | 4 | 5 | 6 | 7 | 8 | 9 |

Scoring: This is Vealey's Trait Sport-Confidence Inventory (TSCI), a measure of the degree of certainty that individuals usually possess about their ability to succeed in sport. To see where you stand, simply add up the 13 numbers you circled to obtain a score ranging from a low of 13 to a high of 117.

______ Your TSCI score

# State Sport-Confidence Inventory (SSCI)

Think about how confident you feel *right now* about performing successfully in the upcoming competition.

Answer the questions below based on how confident you feel *right now* about competing in the upcoming contest. Compare your self-confidence to the *most self-confident athlete* you know.

Please answer as you *really* feel, not how you would like to feel. Your answers will be kept completely confidential.

How confident are you *right now* about competing in the upcoming contest? (circle number)

| | Low | | | Medium | | | | | High |
|---|---|---|---|---|---|---|---|---|---|
| **1.** Compare the confidence you feel right now in *your ability to execute the skills necessary to be successful* to the most confident athlete you know. | 1 | 2 | 3 | 4 | 5 | 6 | 7 | 8 | 9 |
| **2.** Compare the confidence you feel right now in *your ability to make critical decisions during competition* to the most confident athlete you know. | 1 | 2 | 3 | 4 | 5 | 6 | 7 | 8 | 9 |
| **3.** Compare the confidence you feel right now in *your ability to perform under pressure* to the most confident athlete you know. | 1 | 2 | 3 | 4 | 5 | 6 | 7 | 8 | 9 |
| **4.** Compare the confidence you feel right now in *your ability to execute successful strategy* to the most confident athlete you know. | 1 | 2 | 3 | 4 | 5 | 6 | 7 | 8 | 9 |
| **5.** Compare the confidence you feel right now in *your ability to concentrate we1 enough to be successful* to the most confident athlete you know. | 1 | 2 | 3 | **4** | **5** | **6** | **7** | 8 | 9 |
| **6.** Compare the confidence you feel right now in *your ability to adapt to different competitive situations and still be sucful* to the most confident athlete you know. | 1 | 2 | 3 | **4** | **5** | **6** | **7** | 8 | 9 |
| **7.** Compare the confidence you feel right now in *your ability to achieve your competitive goals* to the most confident athlete you know. | 1 | 2 | 3 | **4** | 5 | **6** | **7** | 8 | 9 |
| 8. Compare the confidence you feel right now in *your ability to be successful* to the most confident athlete you know. | 1 | 2 | 3 | **4** | 5 | **6** | **7** | 8 | 9 |
| 9. Compare the confidence you feel right now in *your ability to think and respond successfully during competition* to the most confident athlete you know. | 1 | 2 | 3 | **4** | 5 | **6** | **7** | 8 | 9 |
| 10. Compare the confidence you feel right now in *your ability to meet the challenge of competition* to the most confident athlete you know. | 1 | 2 | 3 | **4** | **5** | **6** | **7** | 8 | 9 |
| 11. Compare the confidence you feel right now in *your ability to be successful based on your preparation for this event* to the most confident athlete you know. | 1 | 2 | 3 | **4** | **5** | **6** | **7** | **8** | **9** |
| 12. Compare the confidence you feel right now in *your ability to perform consistently enough to be successful* to the most confident athlete you know. | 1 | 2 | 3 | **4** | **5** | **6** | **7** | 8 | 9 |
| 13. Compare the confidence you feel right now in *your ability to bounce back from performing poorly and be successful* to the most confident athlete you know. | 1 | 2 | 3 | **4** | **5** | **6** | **7** | 8 | 9 |
