# Supplementary material for: Anthropometric, Physiological, and Psychological Variables That Determine the Elite Pistol Performance of Women
Source: Int J Environ Res Public Health. 2022 Jan 19;19(3):1102. doi: 10.3390/ijerph19031102 (PMC8834022; doi:10.3390/ijerph19031102)
Supplement: Supplementary file 1 [file ijerph-19-01102-s001.zip › ijerph-1510646-supplementaryS2.pdf]

[illegible]	Acsi-28 سوال	[illegible]	[illegible]	[illegible]	[illegible]
1	اهداقی خاصی را به صورت روزانه یا هفتگی تعیین میکنم که هدایتم میکنند.	1	2	3	4
2	من از بیشتر استعداد ها و مهارت هایم استفاده میکنم.	1	2	3	4
3	هنگامی که مربی نحوه اصلاح اشتباهی که مرتکب شده ام را می-گوید، به خودم میگیرم و ناراحت می شوم.	1	2	3	4
4	زمانی که ورزش میکنم، می توانم توجه ام را متمرکز کنم و جلوی حواس پرتی را بگیرم.	1	2	3	4
5	در حین بازی امید و اشتیاق خودم را حفظ میکنم و مهم نیست که چه چیزهای بدی رخ بدهند.	1	2	3	4
6	تحت فشار بهتر بازی میکنم، چون دقیقتر تصمیم میگیرم.	1	2	3	4
7	از اینکه دیگران چطور عملکردم را ارزیابی می کنند کمی نگران هستم.	1	2	3	4
8	برای دستیابی به اهدافم تمایل به برنامه ریزی گسترده ای دارم.	1	2	3	4
9	مطمئنم که خوب بازی خواهم کرد.	1	2	3	4
10	انتقاد مربی یا مدیر از من به جای اینکه کمک کند، ناراحتم می کند.	1	2	3	4
11	هنگام نگاه کردن یا گوش کردن به چیزی، به راحتی می توانم جلوی حواسپرتی خودم را بگیرم.	1	2	3	4
12	با نگرانی از اینکه عملکردم چگونه خواهد بود، فشار زیادی به خودم وارد می کنم.	1	2	3	4
13	برای هر تمرین اهداف عملکردی تنظیم می کنم.	1	2	3	4
14	نیاز ندارم کسی مرا به بازی یا تمرین سخت ترغیب کند، خودم نهایت تلاشم را میکنم.	1	2	3	4
15	اگر مربی در مورد عملکردم انتقادی کند یا سرم فریاد بکشد، من بدون ناراحتی آنها را میپذیرم.	1	2	3	4
16	من شرایط غیر منتظره در ورزش را به خوبی مدیریت میکنم.	1	2	3	4
17	زمانی که در شرایط بدی هستم، به خودم میگویم آرامش خودت را حفظ کن و این کار به من کمک میکند.	1	2	3	4
18	وجود فشار بالا در طول بازی، باعث می شود لذت بیشتری ببرم.	1	2	3	4
19	در حین رقابت، نگران شکست و عملکرد اشتباه خودم هستم.	1	2	3	4
20	پیش از شروع بازی، برنامه ای برای بازی در ذهنم طراحی می-کنم.	1	2	3	4

21	زمانی که احساس تنش بدنی می کنم می توانم بدنم را آرام کنم و به خودم آرامش دهم.	1	2	3	4
22	برای من شرایط پرفشار بعنوان چالش هایی هستند که دوستشان دارم.	1	2	3	4
23	به آنچه که در صورت باختم اتفاق خواهد افتاد، فکر می کنم و آنها را تصور میکنم.	1	2	3	4
24	صرف نظر از اینکه شرایط برای من چگونه است کنترل هیجانم را حفظ میکنم.	1	2	3	4
25	برای من تمرکز روی یک شیء یا یک فرد خیلی آسان است.	1	2	3	4
26	شکست خوردن در رسیدن به اهدافم باعث می شود تا سخت تر تلاش کنم.	1	2	3	4
27	برای بهبود مهارت هایم با دقت به توصیه ها و آموزش ها و دستورالعمل مربیان گوش می دهم.	1	2	3	4
28	به دلیل اینکه در شرایط پرفشار بهتر تمرکز می کنم، کمتر دچار اشتباه میشوم.	1	2	3	4

ردیف	sport confidenceسوال	پاسخ								
		کم			متوسط			زیاد		
		1	2	3	4	5	6	7	8	9
1	وضعیت اعتماد بنفس خود در توانایی انجام مهارت های ورزشی مورد نیاز برای موفقیت در رقابت های ورزشی را با ورزشکاری که به نظر شما در این توانایی دارای اعتماد بنفس بالایی است،مقایسه کنید.									
2	وضعیت اعتماد بنفس خود در توانایی تصمیم گیری های مهم و حساس در رقابت های ورزشی را با ورزشکاری که به نظر شما در این توانایی دارای اعتمادبنفس بسیار بالایی است،مقایسه کنید.									
3	وضعیت اعتماد بنفس خود در توانایی انجام عملکرد ورزشی، تحت شرایط فشار رقابت را با ورزشکاری که به نظر شما دراین توانایی دارای اعتمادبنفس بسیار بالایی است،مقایسه کنید.									
4	وضعیت اعتماد بنفس خود در توانایی عملی کردن نقشه ها بطور موفقیت آمیز را با ورزشکاری که به نظر شما در این توانایی دارای اعتمادبنفس بالایی است،مقایسه کنید.									
5	وضعیت اعتماد بنفس خود در توانایی تمرکز کافی برای موفقیت را با ورزشکاری که به نظر شما در این توانایی دارای اعتمادبنفس بالایی است،مقایسه کنید.									
6	وضعیت اعتماد بنفس خود در توانایی سازگاری با شرایط متفاوت مسابقه را با ورزشکاری که به نظر شما در این توانایی دارای اعتمادبنفس بالایی است،مقایسه کنید.									
7	وضعیت اعتماد بنفس خود در توانایی رسیدن به اهدافی که در رقابتها به آن توجه دارید را با ورزشکاری که به نظر شما در این توانایی دارای اعتمادبنفس بالایی است،مقایسه کنید.									
8	وضعیت اعتماد بنفس خود در توانایی رسیدن به موفقیت در رقابتها را با ورزشکاری که به نظر شما در این توانایی دارای اعتمادبنفس بالایی است،مقایسه کنید.									

9	وضعیت اعتماد بنفس خود در توانایی رسیدن به موفقیت کامل در رقابتها را با ورزشکاری که به نظر شما در این توانایی دارای اعتمادبنفس بالایی است،مقایسه کنید.									
10	وضعیت اعتماد بنفس خود در توانایی تفکر و داشتن عکس العمل مناسب در رقابتهای ورزشی را با ورزشکاری که به نظر شما در این توانایی دارای اعتمادبنفس بالایی است،مقایسه کنید.									
11	وضعیت اعتماد بنفس خود در توانایی روبرو شدن با ماهیت مبارزه جویی رقابت را با ورزشکاری که به نظر شما در این توانایی دارای اعتمادبنفس بالایی است،مقایسه کنید.									
12	وضعیت اعتماد بنفس خود در توانایی رسیدن به موفقیت در رقابتها حتی وقتی که امتیازات شما کمتر از حریفتان است را با ورزشکاری که به نظر شما در این توانایی دارای اعتمادبنفس بالایی است،مقایسه کنید.									
13	وضعیت اعتماد بنفس خود در توانایی بهبود عملکرد ضعیف و سپس رسیدن به موفقیت را با ورزشکاری که به نظر شما در این توانایی دارای اعتمادبنفس بالایی است،مقایسه کنید.									
14	وضعیت اعتماد بنفس فعلی خود در توانایی انجام مهارتهای ورزشی مورد نیاز برای موفقیت در رقابت ورزشی که پیش رو دارید را با ورزشکاری که به نظر شما در این توانایی دارای اعتمادبنفس بسیار بالایی است، مقایسه کنید.									
15	وضعیت اعتماد بنفس فعلی خود در توانایی تصمیم گیری های مهم و حساس در رقابت ورزشی که پیش رو دارید را با ورزشکاری که به نظر شما در این توانایی دارای اعتمادبنفس بسیار بالایی است، مقایسه کنید.									
16	وضعیت اعتماد بنفس فعلی خود در توانایی اجرای عملکرد ورزشی تحت فشار رقابت ورزشی که پیش رو دارید را با ورزشکاری که به نظر شما در این توانایی دارای اعتمادبنفس بسیار بالایی است، مقایسه کنید.									
17	وضعیت اعتماد بنفس فعلی خود در توانایی عملی کردن نقشه ها بطور موفقیت آمیز در رقابت ورزشی که پیش رو دارید را با ورزشکاری که به نظر شما در این توانایی دارای اعتمادبنفس بسیار بالایی است، مقایسه کنید.									
18	وضعیت اعتماد بنفس فعلی خود در توانایی تمرکز کافی برای موفقیت در رقابت ورزشی که پیش رو دارید را با ورزشکاری که به نظر شما در این توانایی دارای اعتمادبنفس بسیار بالایی است، مقایسه کنید.									
19	وضعیت اعتماد بنفس فعلی خود در توانایی سازگاری با شرایط متفاوت رقابت ورزشی که پیش رو دارید را با ورزشکاری که به									

	نظر شما در این توانایی دارای اعتمادبنفس بسیار بالایی است، مقایسه کنید.									
20	وضعیت اعتماد بنفس فعلی خود در توانایی کسب هدفی که در رقابت آینده به آن توجه دارید را با ورزشکاری که به نظر شما در این توانایی دارای اعتمادبنفس بسیار بالایی است، مقایسه کنید.									
21	وضعیت اعتماد بنفس فعلی خود در توانایی رسیدن به موفقیت در رقابت ورزشی پیش رو دارید را با ورزشکاری که به نظر شما در این توانایی دارای اعتمادبنفس بسیار بالایی است، مقایسه کنید.									
22	وضعیت اعتماد بنفس فعلی خود در توانایی تفکر و داشتن عکس العمل مناسب در رقابت ورزشی که پیش رو دارید را با ورزشکاری که به نظر شما در این توانایی دارای اعتمادبنفس بسیار بالایی است، مقایسه کنید.									
23	وضعیت اعتماد بنفس فعلی خود در توانایی روبرو شدن با ماهیت مبارزه جویی در رقابت ورزشی پیش رو دارید را با ورزشکاری که به نظر شما در این توانایی دارای اعتمادبنفس بسیار بالایی است، مقایسه کنید.									
24	بر اساس آماده سازی که برای این مسلبقه داشته اید وضعیت اعتماد بنفس فعلی خود برای رسیدن به موفقیت را با ورزشکاری که به نظر شما در این توانایی دارای اعتمادبنفس بسیار بالایی است، مقایسه کنید.									
25	وضعیت اعتماد بنفس فعلی خود در توانایی رسیدن به موفقیت کامل و صددرصد در رقابت ورزشی که پیش رو دارید را با ورزشکاری که به نظر شما در این توانایی دارای اعتمادبنفس بسیار بالایی است، مقایسه کنید.									
26	وضعیت اعتماد بنفس فعلی خود در توانایی بهبود عملکرد ضعیف و سپس رسیدن به موفقیت در رقابت ورزشی که پیش رو دارید را با ورزشکاری که به نظر شما در این توانایی دارای اعتمادبنفس بسیار بالایی است، مقایسه کنید.									

ردیف	عبارتsport anxiety	اصلا	کمی	در حد متوسط	بسیار زیاد
1	احساس نا ارامی میکنم (عصبی می شوم).				
2	افکار نامربوطی به ذهنم هجوم می آورند.				
3	اعتماد بنفسم را از دست میدهم.				

4	در بدنم احساس تنش میکنم.				
5	نگران میشوم که مبادا به آن خوبی که میتوانم عمل نکنم.				
6	در طول مسابقه فکرم منحرف می شود.				
7	در هنگام مسابقه، نمیتوانم افکارم را بر آنچه اتفاق می افتد متمرکز کنم.				
8	در دلم احساس آشوب میکنم.				
9	در طول مسابقه ، فکر اینکه عملکرد مطلوبی نداشته باشم مانع تمرکز من می شود.				
10	از این نگرانم که مبادا فشار روانی من را از پا درآورد.				
11	ضربان قلبم بالا میرود.				
12	دلم می ریزد.				
13	از این نگرانم که مبادا ضعیف عمل کنم.				
14	به دلیل اضطراب، دچار وقفه های کوتاهی در تمرکز می شوم.				
15	گاهی اوقات قبل از شروع مسابقه یا در حین آن دچار لرزش می شوم.				
16	از اینکه شاید به اهدافم نرسم نگران می شوم.				
17	بدنم خشک و انعطاف ناپذیر می شود.				
18	نگرانم که مبادا دیگران از عملکرد ضعیف من ناامید و ناراحت شوند.				
19	قبل از شروع مسابقه یا در حین آن دچار دلشوره می شوم.				
20	از این نگران می شوم که مبادا نتوانم تمرکز کنم.				
21	قبل از شروع مسابقه دچار تپش قلب می شوم.				

ردیف	نحوه ی امتیاز دهیSms-6	به هیچ وجه	کمی		درحد معمول	خیلی زیاد		کاملا
		1	2	3	4	5	6	7
	بنابر کدامیک از دلایل زیر در این رشته ورزشی فعالیت میکنید:							
1	بخاطر حس هیجانی که به هنگام درگیری جدی در این فعالیت بمن دست میدهد.	1	2	3	4	5	6	7
2	بخاطر اینکه بخشی از راهی است که برای زندگی ام انتخاب کرده ام.							
3	برای اینکه ورزش راه خوبی برای یادگرفتن چیزهای زیادی است که میتوانند در جنبه های دیگر زندگی ام مفید باشند.							
4	بخاطر اینکه بمن اجازه میدهد تا در نظر افرادی که میشناسمشان خوب جلوه کنم.							
5	تا جایی که میدانم، احساس میکنم قابلیت موفقیت در این ورزش را ندارم.							
6	چون هنگامی که بر تکنیک های سخت تسلط می یابم، احساس رضایتمندی بیشتری دارم.							
7	برای اینکه کسی که می خواهد از لحاظ بدنی روی فرم بماند، انجام ورزش حتما لازم است.							
8	برای اینکه یکی از بهترین راه هایی است که برای پرورش دیگر ابعاد زندگی خود انتخاب کرده ام.							
9	چون جزئی است که بمن متصل شده است.							
10	برای اینکه باید ورزش کنم تا احساس خوبی از خود داشته باشم.							
11	برای کلاس(پرستیژ) که ورزشکار بودن برایم دارد.							
12	مطمئن نیستم که دیگر بخواهم وقت و تلاشم را به هر شکلی در رشته ورزشی ام هدر دهم.							
13	بخاطر اینکه شرکت در رشته ورزشی ام با اصول اخلاقی ام مطابقت دارد.							
14	برای رضایتی که هنگام کامل کردن توانایی هایم تجربه میکنم.							
15	برای اینکه این یکی از بهترین راههای حفظ روابط خوب با دوستانم است.							

16	اگر برای ورزش وقت کافی نگذارم، حس بدی بمن دست میدهد.							
17	زیاد برایم روشن نیست، واقعا فکر نمیکنم جای من در ورزش باشد.							
18	بخاطر لذتی که از کشف راهبردهای اجرایی بمن دست میدهد.							
19	بخاطر منافع مادی یا اجتماعی که ورزشکار بودن دارد.							
20	چون تمرین سخت عملکرد من را بهبود خواهد بخشید.							
21	چون شرکت در رشته ورزشی ام، جزء جدایی ناپذیر زندگی ام است.							
22	بنظر می رسد که مثل سابق از رشته ورزشی ام لذت نمیبرم.							
23	برای اینکه باید بطور منظم ورزش کنم.							
24	برای اینکه به دیگران نشان دهم به چه خوبی در رشته ورزشی ام شایسته هستم.							
